# Sources of implicit and explicit intergroup race bias among African-American children and young adults

**DOI:** 10.1371/journal.pone.0183015

**Published:** 2017-09-28

**Authors:** Bentley L. Gibson, Philippe Rochat, Erin B. Tone, Andrew S. Baron

**Affiliations:** 1 Department of Psychology, Georgia Highlands College, Rome, Georgia, United States of America; 2 Department of Psychology, Emory University, Atlanta, Georgia, United States of America; 3 Department of Psychology, Georgia State University, Atlanta, Georgia, United States of America; 4 Department of Psychology, University of British Columbia, Vancouver, British Columbia, Canada; University of California Berkeley, UNITED STATES

## Abstract

Implicit intergroup bias emerges early in development, are typically pro-ingroup, and remain stable across the lifespan. Such findings have been interpreted in terms of an automatic ingroup bias similar to what is observed with minimal groups paradigms. These studies are typically conducted with groups of high cultural standing (e.g., Caucasians in North America and Europe). Research conducted among culturally lower status groups (e.g., African-Americans, Latino-Americans) reveals a notable absence of an implicit ingroup bias. Understanding the environmental factors that contribute to the absence of an implicit ingroup bias among people from culturally lower status groups is critical for advancing theories of implicit intergroup cognition. The present study aimed to elucidate the factors that shape racial group bias among African-American children and young adults by examining their relationship with age, school composition (predominantly Black schools or racially mixed schools), parental racial attitudes and socialization messages among African-American children (N = 86) and young adults (N = 130). Age, school-type and parents’ racial socialization messages were all found to be related to the strength of pro-Black (ingroup) bias. We also found that relationships between implicit and explicit bias and frequency of parents' racial socialization messages depended on the type of school participants attended. Our results highlight the importance of considering environmental factors in shaping the magnitude and direction of implicit and explicit race bias among African-Americans rather than treating them as a monolithic group.

## Introduction

Biases to generate positive evaluations of people affiliated with one’s own group have been well documented as normative in the literature on intergroup cognition and its putative evolutionary roots [1]. According to Social Identity Theory [2–3] and Social Identity Development Theory [4], such biases stem from a pervasive desire to feel positive about oneself based on one’s group memberships. Many studies reveal that ingroup biases across a variety of social categories (e.g., gender, race, nationality, religion, language) emerge by age 6 [5–13]. Even when group membership is arbitrarily determined, as it is in minimal group experiments, elementary school-aged children [14–16] and adults [17–18] show a bias for their novel ingroup over a newly formed outgroup, suggesting that the tendency to prefer one’s own group may follow naturally from identifying with a group. Previous research has reported a positive relationship between ingroup preference and ingroup identity for both children and adults [19–21], further suggesting that self-identification with a particular group is linked to a positive evaluation of that group.

However, there is also evidence that the ingroup bias predicted by Social Identity Theory is not necessarily the norm. According to System Justification Theory [22–23], intergroup bias is not merely shaped by a desire to uplift the ingroup in order to build self-esteem (as postulated by Social Identity Theory), but is also impacted by a motivation to align with the social norms of the existing socio-political structure. According to SJT, individuals will positively evaluate those social groups that are positively portrayed in society, even when those groups are different from one’s ingroup.

A large body of research that has influenced our understanding of the nature of intergroup bias has focused on explicit and implicit measures of race bias [1; 6–8; 10; 21; 23]. Explicit, or conscious, biases are well thought-out responses over which people have a certain level of control [24–28]. They are difficult to interpret in research on intergroup preferences, in part because people try to monitor and control the degree to which they reveal these biases to others [23; 29–39]. Implicit measures of intergroup bias, in contrast, are less obtrusive and less sensitive to suppression because of a desire to “look good”. Implicit measures of intergroup bias are particularly important in social climates where there are strong social norms against expressing negative outgroup attitudes (or stereotypes). Illustrating this contrast between explicit and implicit measures, European-Americans may report explicit pro-ingroup race bias during childhood, but this bias diminishes with age [7–8; 21;32–35], falling in alignment with the social norm of being motivated to appear unbiased. In contrast, European-Americans’ implicit pro-ingroup race bias appears stable through adulthood [7–8].

On both explicit and implicit measures, African-Americans exhibit a different developmental pattern compared to European-Americans. On explicit measures, African-Americans’ pro-ingroup race bias *increases* with age, falling more in alignment with the social norm of being motivated to show “Black Pride” [21; 24–25; 40; 41–42]. In contrast, on implicit measures, African-Americans and other culturally stigmatized groups provide notable exceptions to the European-American pattern of showing a stable and consistent pro-ingroup implicit bias. Specifically, among African-American children [40; 43–44] and adults [24–25; 45–46], research shows no mean level preference for either Black or White individuals, with approximately half of the population showing an implicit pro-Black over White bias and the other half revealing an implicit pro-White over Black bias. Comparable findings of a lack of an implicit ingroup bias when the comparison group is White have emerged in studies of youth from other social and ethnic groups, including those who identify as South-African [47] and Hispanic-American [48].

The present study explored several factors that might modulate the direction of implicit race bias among African-American children and young adults. Although we also examined explicit race bias, we were particularly interested in the implicit race bias of African-Americans’ because far less is understood about how bias is shaped. Previous research on European-Americans’ implicit race bias and African-Americans’ explicit race bias [40; 43; 45–46] inspired us to explore how the racial composition of the school that an individual attends, as well as parents’ racial attitudes and transmission of racial socialization messages, may help explain the variability in African-Americans’ implicit race bias.

### School racial composition

McGlothlin and Killen (2010) investigated the impact of school type on the implicit race bias of European-Americans attending racially homogeneous or racially heterogeneous schools. They found that children enrolled in heterogeneous schools had lower pro-White over Black implicit bias than did their counterparts in homogeneous schools [44]. Newheiser and Olson (2012) also examined the role that school racial composition played in shaping children’s implicit race bias [40], and like McGlothlin and Killen (2010) they only looked at the impact of school diversity on European-American children. Using a different measure of implicit race bias, they found no difference in the strength of implicit race bias among European-American children attending racially homogenous or heterogeneous schools, underscoring the need for further work in this area.

In a study focusing on the relationship between school racial composition and explicit bias among African-American 8-11-year-olds [49], Dutton, Singer, and Devlin (1998) revealed that children from predominantly Black schools had stronger pro-Black bias on an explicit measure than their counterparts in integrated or predominantly White schools. This finding suggests that African-American children’s explicit race bias can vary based on their school context. Similar findings have been observed when comparing African-Americans from predominantly White colleges to those from Historically Black Colleges (HBC), with those attending HBCs revealing stronger explicit pro-Black bias [50]. In our study, we sought to extend this line of work by examining how school racial composition relates to implicit race bias in a sample of African-American children and young adults.

### Parent attitudes and messages about race

There is no research on the impact of African-American parents’ racial attitudes on their children’s implicit race bias, but previous work on European-Americans has found a significant relationship between parents’ racial attitudes and their children’s implicit race bias [51]. However, there is previous research on the relationship between African-American parents’ racial attitudes and behaviors on their children’s *explicit* race bias that has informed the current study. Thornton (1997) characterized racial socialization among African-Americans as the practice of conveying messages about positive self-image, understanding discrimination based on race, and acceptance of being African-American. Further, the practice involves an emphasis on Black history and racial pride [52]. Approximately two-thirds of African-American parents report transmitting some form of racial socialization messages to their children [53]. Spencer (1983) examined the association between African-American mothers’ transmission of cultural values and their children’s explicit, pro-Black over White bias [54]. In this study, the author interviewed parents about the cultural messages they conveyed to their children (e.g., discussion of the Civil Rights era) and also about their own cultural attitudes. Results showed that the mothers’ pro-Black bias and their frequency of initiating discussions about Black history predicted children’s explicit pro-Black over White bias. These findings are of particular relevance to the current study because emphasizing positive information about cultural history and heritage has been link to children’s explicit pro-Black over White bias [54–60]. The present study extends this work by examining associations between African-American children’s *implicit* race bias and their parents’ attitudes and messages about race.

### Present study

Based on the literatures reviewed above, we designed a cross-sectional study comparing patterns of implicit race bias among African-American children and young adults who attended different school types (racially homogeneous vs. racially heterogeneous). Specifically, we examined relationships among both implicit and explicit measures of race bias and age, school racial composition, parents’ racial attitudes, and the racial socialization messages that parents conveyed to their children. Because this is the first study to examine multiple factors associated with African-American children and young adults’ implicit race bias, it is an exploratory analysis.

## Materials and methods

### Ethics statement

This study was approved by the authors, as well as by Emory University's Institutional Review Board (#IRB00053238). Written informed consent was obtained from the adult participants and from the parents of child participants.

### Participants

A total of 216 African-American people from two age groups participated in this study: 86 children constituted the child, primary school sample (*M* = 8.73 years, *SD* = 2.59 years, females = 48) and 130 young adult participants constituted the older sample (*M* = 19.75 years, *SD* = 1.23 years, females = 104). The child sample was recruited from a university database of families interested in research participation for children, as well as from local elementary schools within the Greater-Atlanta area. Parents were asked to report if their children attended either predominantly Black/homogeneous schools (90% or more Black), or racially integrated/heterogeneous schools (Black population between 10% and 50%). Young adult participants were recruited from a Historically Black (over 90% African-American), all-female college and from three racially heterogeneous universities (10–20% African-American). Although the majority of our young adult sample was female, previous research has found no evidence of gender differences in implicit race bias [7–8; 48]. Approximately half of the sample (48%) attended predominantly Black/homogeneous schools; the remaining participants attended racially integrated/heterogeneous schools. Child participants received a small toy as a token of appreciation for their participation and college-aged participants received course credit for their participation. All participants in the study either self-identified as African-American or their parents identified them as such.

We also collected socio-economic status (SES) information from our young adult sample and from parents of our child sample using the Hollingshead Four Factor Scale [61]. Parents of 82 participating children (46 homogeneous schools, 36 heterogeneous schools) provided this information, as did 124 young adult participants (52 homogeneous school, 72 heterogeneous schools). This information allowed us to calculate a total SES score for each participant based on mother and father educational and occupational level. There were no school differences in total socio-economic status (SES) scores for children, t (80) = -.77, *p* = .44, or young adults, t (122) = .26, *p* = .79.

### Measures

#### Implicit tasks

The Child Implicit Association Test [7–8; 48] was used to measure implicit race bias and group identity for all participants. The bias and identity measures were designed using stimuli and parameters described in previous research with children and young adults [7; 40; 48]. To measure implicit race bias, participants were first instructed to classify images of child faces (half males and half females) into one of two categories (European-American and African-American) using two large response buttons that were color matched with category reminders on the screen. We used images that have been employed in previous cross-sectional studies of implicit race bias [7; 40; 48]. For 20 trials, images of European-American and Africa-American faces appeared one at a time on the screen, and participants had to determine which race that face belonged to by pressing the corresponding button, after which the task advanced to the next face. Next, participants practiced categorizing evaluative words as either good or bad using the same two response buttons for 20 trials (e.g., participants were instructed to press the yellow button for good words and to press the blue button for bad words). The words used (*happy*, *nice*, *fun*, *good*, *bad*, *mean*, *yucky*) were presented acoustically (with a female voice) for children and adults, just as they were in prior studies.

Following these two warm-up tasks, participants were presented with a block of 40 trials during which they were told that they would see pictures of faces and hear words, one at a time. They were instructed to press the button that appropriately categorized each stimulus, just as they had done previously during the warm-up tasks (e.g., press the yellow button in response to African-American faces or good words and press the blue button in response to European-American faces or bad words). Faces and words were presented on alternating trials, but the exemplar order was random. After completing these trials, participants practiced categorizing the same series of faces according to race (African-American/European-American) again. In this trial block, however, the corresponding categories changed sides (i.e., button reversal) for a total of 40 trials. Next, participants were presented with a another block of test trials (40 in total) in which they once again were instructed to categorize faces and words using the same two response keys. This time participants were asked to press the yellow button in response to African-American faces or bad words and to press the blue button in response to European-American faces or good words. Speed and accuracy were recorded for each block of trials. Trials only advanced following a correct response. Participants’ IAT scores were calculated by subtracting the mean response latency for own-race incompatible trials (African-American + bad, European-American + good) from the mean response latency for own-race compatible trials (African-American + good, European-American + bad). For half of the participants, “African-American” was first paired with good words and “European-American” was paired with bad words. For the other half of participants “African-American” was first paired with bad words and “European-American” was paired with good words.

To measure implicit race identity, we used the procedure described above, with one change. The positive and negative attributes (i.e., good and bad words) were replaced by self-relevant words (*me*, *I*, *myself*, *and my*) and other-relevant words (*them*, *their*, *themselves*, *and they*) [19; 48]. Thus, implicit ingroup identity was measured by comparing participants’ speed and accuracy when pairing African-American faces with self-relevant versus other-relevant words to their speed and accuracy when pairing European-American faces with the same set of words. Following procedures recommended by Greenwald et al., [20] and adapted for use with child and adolescent samples [7–8; 48], each participant’s implicit identity score was calculated by subtracting the mean response latency for ingroup identity incompatible trials (African-American + other relevant words, European-American + self-relevant words) from the mean response latency for ingroup identity compatible trials (African-American + self-relevant words, European-American + other-relevant words).

The resulting differences for each task (attitude and identity) were then divided by the pooled standard deviation for both compatible and incompatible blocks of trials. The data were computed so that scores greater than 0 indicate an implicit pro-Black over White bias/identity, and scores less than 0 indicate an implicit pro-White over Black bias/identity. All participants completed the Race-IAT first, followed by the Identity-IAT.

#### Explicit tasks

The explicit race bias and identity measures were based on tasks used in past research with similarly-aged samples [48]. All explicit measures were administered after the implicit tasks in each session. For the Explicit Race Bias task, participants were presented with four trials in which the face of an African-American individual appeared next to the face of a European-American individual (matched for sex) and participants were instructed to indicate which person they liked most. For the Explicit Race Identity task, participants were presented with an additional four sets of trials and were asked to indicate which individual was most like themselves. The same eight faces used in prior research (four African-American, four European-American) were used for both the bias and identity tasks [48]. The side of the screen on which each face of each race appeared was counterbalanced within each task (bias and identity). Separate scores were derived for the explicit bias and explicit identity measures. For each measure, we calculated the percentage of trials participants chose the African-American face over the European-Americans face.

#### Adults’ ethnic identity measure

Young adult participants and parents of the children in the younger sample completed the Multi-group Ethnic Identity Measure (MEIM) [10], a 23-item questionnaire probing 4 aspects of ethnic identity: positive ethnic attitudes and sense of belonging, other-group attitudes, ethnic identity achievement (learning positive information about African-Americans), and ethnic behaviors (e.g., participation in positive ethnic activities such as church, cultural organizations, etc.). Participants rated their agreement with each item on a four-point Likert scale ranging from 1 = *strongly disagree* to 4 = *strongly agree*. Ratings were also summed to yield a total score; higher scores are interpreted as indicating a stronger ingroup racial identity.

#### Parents’ racial socialization measure

The Parental Racial Socialization questionnaire [58] was used to measure the frequency with which parents transmitted different types of messages about race to their children. Parents were asked to estimate on a 6-point scale (0 = *none* to 5 = *more than seven times*) how many times over the preceding 12 months they expressed three kinds of messages about race to their children. The first category involves teaching cultural pride and facts relevant to Black history (*Cultural Socialization)*, the second category involves teaching about discrimination (*Preparation for Bias)*, and the third category involves warning their children not to trust other groups (*Promotion of Mistrust)*. A separate total score was calculated for each category.

### Procedure

A female African-American experimenter administered all tests and questionnaires to all participants individually, either in a university laboratory or onsite at participating schools. After obtaining written consent from either the adult participants or parents of child participants, each participant sat in front of a table, facing a computer monitor placed 2 feet away. Measurement type order (implicit then explicit) was fixed, similar to past research [7–8; 40; 48], but task order within each measurement type (bias and identity) was randomized across participants of both age groups. Parents of participating children were asked to complete the MEIM followed by the Racial Socialization questionnaire. College students were asked to complete the MEIM following completion of implicit and explicit bias and identity measures.

## Results and discussion

Data were first examined for accuracy of data entry, missing values, and fit between their distributions and the assumptions of multivariate analysis. As is customary in studies of implicit attributions, data from participants with error rates exceeding 30%, or those for whom more than 30% of the IAT trials recorded response latencies less than 300ms, were excluded from analyses [19]. These criteria resulted in the exclusion of 7 participants’ implicit bias data (6 children and 1 young adult) and 18 participants’ implicit identity data (17 children and 1 young adult), yielding 210 usable cases for the implicit bias measure and 199 usable cases for the implicit identity measure.

### Implicit bias

**Fig 1:**
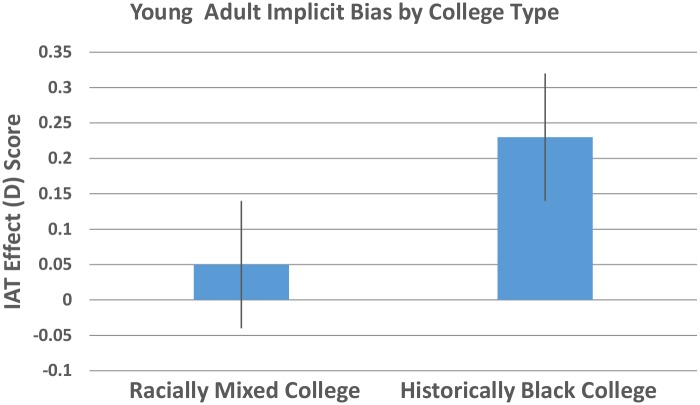
Mean IAT scores by college type; Scores above zero reflect an implicit pro-Black over White bias; Scores below zero reflect an implicit pro-White over Black bias. Error bars represent the standard error of the mean.

Results of a one-sample t-test in which we compared the average IAT scores to zero (no implicit bias) for each age group indicated a significant implicit pro-Black over White bias among young adults, *M* = .12, *SD* = .68, *SE* = .06, *CI* [-.01, .24], *t* (129) = 2.07, *p* = .04, *d* = .36. This implicit pro-Black over White bias in young adults was only found among individuals attending the Historically Black College, *M* = .23, *SD* = .62, *SE* = .09, *CI* [.06, .40], *t* (52) = 2.68, *p* = .01, *d* = .72. Their counterparts attending a racially heterogeneous college did not exhibit a significant implicit bias for either their racial ingroup or outgroup, *M* = .05, *SD* = .73, *SE* = .09, *CI* [-.12, .22], *t* (71) = .61, *p* = .55 (Fig 1, S1 File). Interestingly, only those young adults who attended predominantly Black schools from K-12 showed an implicit pro-Black over White bias, *M* = .21, *SD* = .57, *SE* = .09, *CI* [.02, .39], *t* (38) = 2.29, *p* = .03, *d* = .74. This was not the case for those who attended racially heterogeneous schools from K-12, *M* = .08, *SD* = .74, *SE* = .05, *CI* [-.08, .24], *t* (82) = 1.01, *p* = .31.

**Fig 2:**
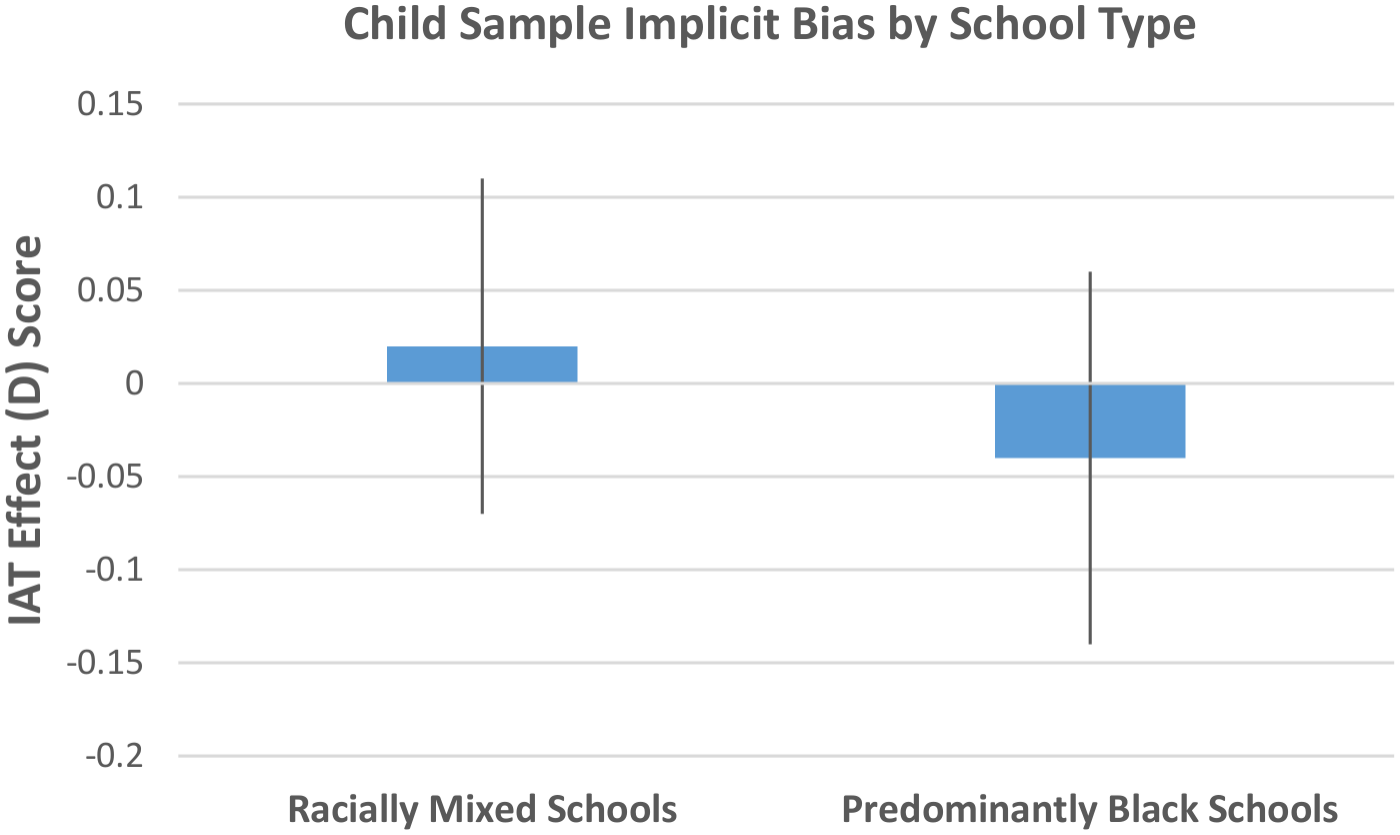
Mean IAT scores by school type; Scores above zero reflect an implicit pro-Black over White bias; Scores below zero reflect an implicit pro-White over Black bias. Error bars represent the standard error of the mean.

By contrast, members of the child sample showed no evidence of an implicit bias for either the ingroup or the outgroup, *M* = -.03, *SD* = .63, *SE* = .07, *CI* [-.17, .11], *t* (79) = -.37, *p* = .71. Furthermore, there were no observed differences in implicit bias among children attending homogeneous schools (*M* = -.04, *SD* = .67, *SE* = .10) and those attending heterogeneous schools (*M* = .02, *SD* = .56, *SE* = .09), *t* (81) = -.48, *p* = .64 (Fig 2, S2 File).

**Fig 3:**
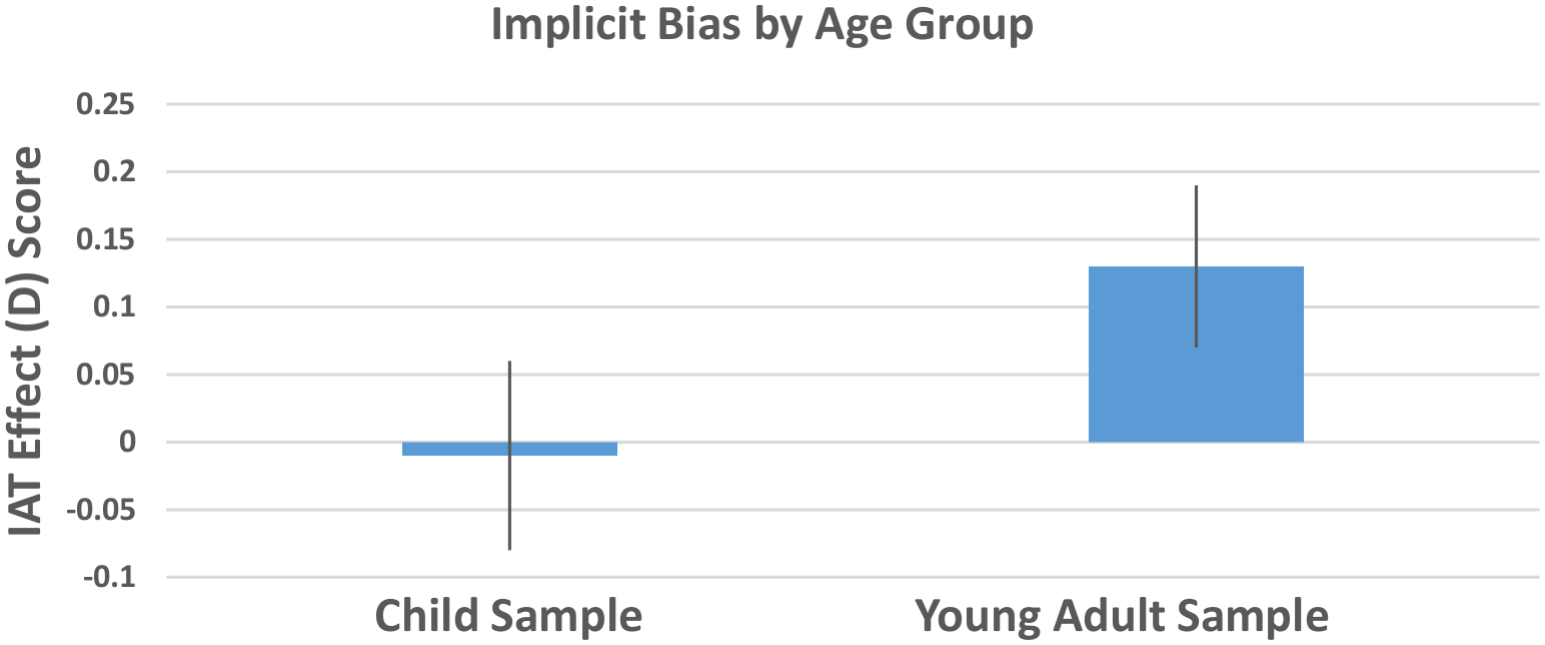
Mean IAT scores among the child sample and the young adult sample. Scores above zero reflect an implicit pro-Black over White bias; scores below zero reflect an implicit pro-White over Black bias. Error bars represent the standard error of the mean.

A 2 (age group) x 2 (school type) ANOVA with implicit bias IAT score as the dependent variable revealed a non-significant overall model, *F* (3, 210) = 1.66, *p* = .18, η^2^_p_ = .02. There was a trend toward a main effect of age group with the young adult sample (*M* = .12, *SD* = .68, *SE* = .06) having stronger implicit pro-Black bias than the child sample (*M* = -.03, *SD* = .63, *SE* = .07), *F*(1, 209) = 2.95, *p* = .09, η^2^_p_ = .01 (Fig 3, S3 File). There was no main effect of school type or interaction between age group and school type (all *p*’s > .27).

### Explicit bias

A one sample t-test was computed to compare the mean percent of times participants chose a Black face over a White face on the four trials (0%, 25%, 50%, 75%, or 100%). Scores were compared to 50% (indicating no explicit preference for either Black or White faces). A majority of both the child sample, *M* = 65.70%, *SD* = 32.10, *SE* = 3.46, *t* (85) = 4.54, *p* < .01, *d* = .98, and the young adult sample, *M* = 78.46%, *SD* = 24.66, *SE* = 2.16, *t* (129) = 13.16, *p* < .01, *d* = 2.31, exhibited an explicit pro-Black preference, choosing the Black face over the White face significantly more often as the one they “*liked the most*”.

A 2 (age group) x 2 (school type) ANOVA with explicit bias scores as the dependent variable revealed a significant model, *F* (3, 216) = 4.47, *p*< .01, *η*^*2*^_p_ = .06. There was a main effect of age group, with the young adult sample (*M* = 78.46%, *SD* = 24.66, *SE* = 2.16) choosing the Black face over the White face on the explicit bias measure more often than did the child sample (*M* = 65.70%, *SD* = 32.10, *SE* = 3.46), *F*(1, 216) = 11.17, *p <* .01, η^2^_p_ = .05. There was no main effect of school type, or interaction between age group and school type (all *p*’s > .14).

### Implicit identity

**Fig 4:**
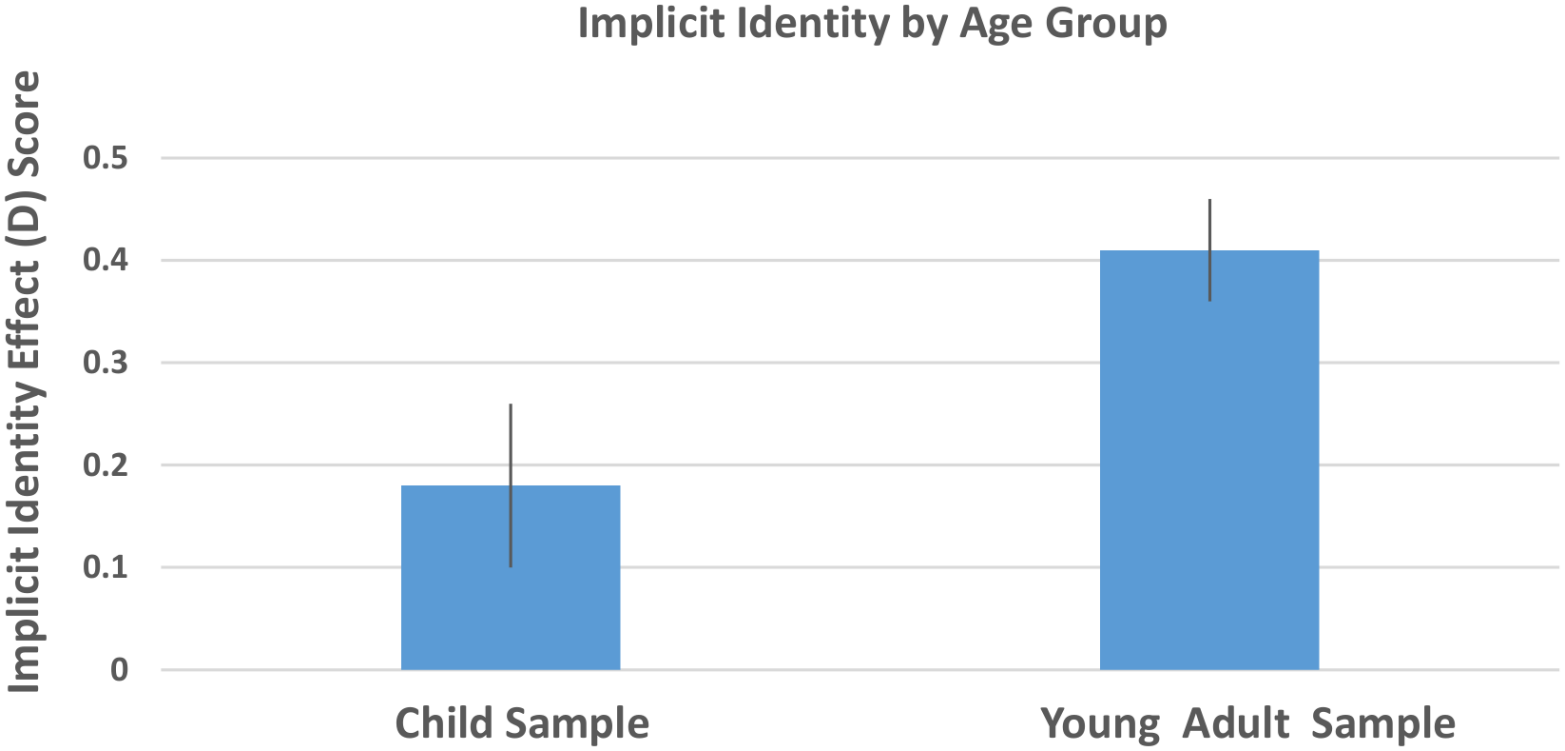
Mean implicit identity scores among the child sample and the young adult sample. Scores above zero reflect an implicit Black over White identity; scores below zero reflect an implicit pro-White over Black identity. Error bars represent the standard error of the mean.

Results of a one-sample t-test in which we compared the average implicit identity scores to zero (neutral implicit identity) for each age group indicated that the child sample, *M* = .18, *SD* = .68, *SE* = .08, *CI’s* [.03, .33], *t* (69) = 2.16, *p* = .03, *d* = .52, and young adult sample, *M* = .41, *SD* = .67, *SE* = .05, *CI’s* [.30, .53], *t* (128) = 7.07, *p* < .01, *d* = 1.25, both exhibited a strong implicit identity with Black over White faces. A 2 (age group) x 2 (school type) ANOVA revealed that young adults (*M* = .41, *SD* = .67, *SE* = .05) had a stronger implicit identity with Black over White than the child sample (*M* = 0.18, *SD* = .68, *SE* = .08), *F*(1,199) = 5.88, *p* = .02, η^2^_p_ = .03 (Fig 4). There was no main effect of school type or interaction between age group and school type (all *p*’s > .59).

### Explicit identity

A one sample t-test compared the mean percent of times participants chose a Black face over a White face on the four trials (0%, 25%, 50%, 75%, or 100%). Scores were compared to 50% indicating no explicit identification with either Black or White faces. Both the child sample, *M* = 84.30%, *SD* = 27.67, *SE* = 2.98, *t* (85) = 11.50, *p* < .01, *d* = 2.49, and the young adult sample, *M* = 91.73%, *SD* = 17.72, *SE* = 1.55, *t* (129) = 26.86, *p* < .01, *d* = 4.73, explicitly identified with Black over White.

A 2 (age group) x 2 (school type) ANOVA with explicit identity scores as the dependent variable revealed a trend towards a significant model, *F* (3, 216) = 2.31, *p* = .08, *η*^*2*^_p_ = .03. There was a main effect of age group, with the young adult sample (*M* = 91.73%, *SD* = 17.72, *SE* = 1.55) choosing the Black face over the White face more often than the child sample (*M* = 84.30%, *SD* = 27.67, *SE* = 2.98), *F*(1, 216) = 5.96, *p* = .02, *η*^*2*^_p_ = .03. There was no main effect of school type or interaction between age group and school type (all *p*’s > .34). (See Table 1 for all age group comparisons on implicit and explicit measures).

**Table 1:** Mean scores on all measures by age group.

	Child Sample		Young Adult Sample	
	M	SD	M	SD
**Implicit Bias**	**-0.03**	**0.63**	**0.12**	**0.68**
**Explicit Bias**	**65.70%**	**32.1**	**78.46%**	**24.66**
**Implicit Identity**	**0.18**	**0.68**	**0.41**	**0.67**
**Explicit Identity**	**84.30%**	**27.67**	**91.73%**	**17.72**

On implicit measures, scores above zero reflect a pro-Black bias/identity; scores below zero reflect a pro-White over Black bias/identity. On explicit measures, scores above 50% indicate a pro-Black over White bias/identity.

### Relationships between parent measures and biases

We found a number of relationships between parent and child measures (Table 2). There was a trend toward a negative relationship between parents’ frequency of exposure to preparation for bias messages and their children’s implicit bias, *r* (76) = —.15, *p* = .10. Specifically, for children from all-Black schools, parents’ frequency of bias preparation messages significantly predicted their children’s implicit bias, *B* = -.51, *t* (40) = -2.22, *p* = .03. This relationship was not significant for participants from heterogeneous schools, *r* (34) = .18, *p* = .15. We also found a trend of a relationship between parents’ ethnic behaviors and their children’s implicit bias, *r*_*s*_ (78) = .18, *p* = .06. Strong ethnic behaviors, such as participating in cultural activities, were only significantly associated with pro-Black over White implicit bias for children who attended heterogeneous schools, *r*_*s*_ (35) = .32, *p* = .03, but not those attending racially homogeneous schools, *r*_*s*_ (43) = .09, p = .29.

**Table 2:** Correlations among all parent and child measures by school type.

Parent Measure	Implicit Bias	Explicit Bias	Implicit Identity	Explicit Identity
	n, *r*, *p*	n, *r*, *p*	n, *r*, *p*	n, *r*, *p*
**Belonging**	78, .13, .12	82, .10, .46	71, -.02, .43	82, .05, .32
Belonging- homogeneous schools	43, .08, .31	45, -.07, .34	40, .06, .35	45, -.06, .34
Belonging- heterogeneous schools	36, .24, .08*	37, .11, .25	31, -.10, .29	37, .28, .05**
**Ethnic identity**	78, .13, .14	82,.-.11, .16	71, -. 06, 32	82, -07, .26
Ethnic identity- homogeneous schools	43, .20, .10*	45, -.09, .29	40, .05, .38	45, -.22, .07*
Ethnic identity- heterogeneous schools	35, .01, .47	37, -.18, .13	31, -.26, .08*	37, .15, .18
**Ethnic behaviors**	**78, .18, .06** *	82,.-.01, .46	71, -.01,.47	82, -.05, .34
Ethnic behaviors- homogeneous schools	43, .09, .29	45, -.10, .26	40, .07, .33	45, .19, .11
Ethnic identity- heterogeneous schools	35, .32, .03**	37, .08, .31	31,-.11, .29	37, .11, .26
**Other-group attitudes**	**78, -.18, .06** *	82, .04, .37	71, -.05, .33	82, .21, .03*
Other-group- homogeneous schools	**43, -.22, .08** *	45, .10, .25	40, -.04, .39	45, .14, .18
Other-group heterogeneous schools	35, -.22, .09*	37, -.05, .38	31, -.06, .37	37, .28, .05**
**MEIM Scores**	**78, .18, .06** *	82, -.10, .20	16, -.02, .42	82, -.08, .24
MEIM- homogeneous schools	43, .19, .11	45, -.09, .28	40, .06, .35	45, -.22, .08*
MEIM- heterogeneous schools	35, .20, .13	37, -.13, .23	31, -.13, .25	37, .04, .41
**Preparation for Bias**	**76, -.15, .10** *	80, .20, .04**	68, .30, .01 **	80, .05, .34
Preparation- homogeneous schools	**42, -.33, .02** **	44, .07, .32	39, .23, .08 *	44, .03, .41
Preparation- heterogeneous schools	34, .18, .15	36, .39, < .01***	29, .35, .03**	36, .07, .34
**Racial Socialization**	76, .05, .34	80, .13, .12	**68, .20, .06** *	80, .10, .18
Racial socialization- homogeneous schools	43, .02, .46	44, .15, .16	**39, .26, .06** *	44, .27, .04**
Racial socialization- heterogeneous schools	34, .13, .24	36, .08, .33	29, .16 .20	36, -.08, .31

* *p* ≤ .10.

** *p* ≤ .05.

*** *p* < .01.

There was a significant positive relationship between the frequency with which parents transmitted preparation for racial bias messages and their children’s explicit pro-Black over White bias, *r*(80) = .20, *p* = .04. Furthermore, there was a trend for parents’ preparation for racial bias messages to predict children’s explicit bias, *B* = .65, *t* (78) = 1.80 *p* = .08. In particular, when participants were divided according to type of school attended, the correlation *r* (36) = .39, *p <* .01, and regression, *B* = 1.40, *t*(34) = 2.50, *p* = .02, were only significant for those who attended heterogeneous schools.

### Relationship between parent measures and identity

We also observed several relationships between parent measures and child identity (Table 2). The frequency with which parents reported sending preparation for bias messages to their children was positively associated with children’s implicit identity, such that children exhibited stronger implicit identification with Black over White faces the more their parents warned them about racism, *r* (68) = .25, *p* = .02. Parents’ frequency of preparation for bias messages significantly predicted children’s implicit race identity, *B* = .45, *t* (66) = 2.11, *p* = .04. This relationship was stronger for participants attending heterogeneous schools, *r* (29) = .35, *p* = .03, than for their counterparts in homogeneous schools, *r* (39) = .21, *p* = .10. There was also a non-significant trend for racial socialization to predict implicit identity, *B* = .14, *t* (66) = 1.91, *p* = .06, such that parents who endorsed emphasizing Black history had children with a marginally stronger implicit Black over White identity. This relationship was marginally significant for those in racially homogeneous schools, *r*_*s*_ (39) = .26, *p* = .06, but non-significant for those in racially heterogeneous schools, *r*_*s*_ (29) = .16, *p* = .20.

We found a significant positive correlation between explicit race identity and parents’ outgroup attitudes for the child sample, *r*_*s*_ (82) = .21, *p* = .03. Specifically, parents who endorsed spending more time with outgroup members had children who identified more strongly with Black over White faces, *B* = .61, *t* (80) = 1.98, *p* = .05. This relationship was only found for children who attended racially heterogeneous schools, *r*_*s*_ (37) = .28, *p* = .05. It also was only a predictor for children in heterogeneous schools, *B* = 1.00, *t* (35) = 2.23, *p* = .03. This relationship was not significant for participants who attended racially homogeneous schools, *r*_*s*_ (45) = .14, *p* = .18.

### Relationship between group bias and group identity

Children’s implicit bias and implicit identity scores were not significantly associated with each other, *r* (74) = .09, *p* = .47; however, there was a significant positive relationship between implicit bias and implicit identity scores for young adults, *r* (125) = .36, *p* < .01. The relationship between implicit bias and implicit identity varied according to participants’ school type. For participants who attended heterogeneous schools, this significant relationship was positive [child sample: *r* (33) = .47, *p* < .01; young adult sample: *r* (72) = .44, *p* < .01]. No significant findings were observed for either age group among participants who attended homogeneous schools.

There was a significant relationship between explicit bias and explicit identity scores for the child sample, *r* (90) = .54, *p* < .01, and the young adult sample, *r* (126) = .48, *p* < .01. For the child sample, the relationship between explicit bias and explicit identity was stronger in the homogeneous school, *r* (48) = .67, *p* < .01, than in the heterogeneous school, *r* (42) = .38, *p* < .01. Similar results were found with the young adult sample in the all-Black college showing a stronger relationship between explicit bias and explicit identity, *r* (53) = .52, *p* < .01, than participants attending a racially heterogeneous college, *r* (73) = .44, *p* < .01.

Young adults’ implicit bias score was significantly associated with both their explicit bias score, *r*(125) = .27, *p* < .01, and their explicit identity score, *r*(125) = .23, *p* = .01. Interestingly, when these associations were examined separately according to type of school attended, the relationship between implicit and explicit bias scores, *r* (72) = .33, *p* < .01, as well as the relationship between implicit bias and explicit identity scores, *r* (72) = .34, p < .01, were significant only for students who attended a racially heterogeneous college. The younger sample’s implicit bias and explicit bias scores were associated at a level that approached, but did not reach, significance, *r*(83) = .20, *p* = .07. The child sample’s implicit bias scores were not significantly associated with explicit identity scores, (*p* = .70) (Table 3).

**Table 3:** Relationships between implicit and explicit measures by school type and age group.

	Implicit Bias and Explicit Bias	Implicit Bias and Explicit Bias2	Implicit Identity and Explicit Bias	Implicit Identity and Explicit Identity
	n, r, *p*	n, r, *p*	n, r, *p*	n, r, *p*
**CHILD SAMPLE**	**83, .20, .07** *	83, -.04, .70	76, .17, .15	76, .07, .55
**Homogeneous**	45, .17, .27	45, -.14, .35	42, .16, .32	42, .09, .56
**Heterogeneous**	38, .25, .13	38, .10, .56	34, .19, .28	34, .04, .80
**YOUNG ADULTS**	125, .27, < .01**	125, .23, *<* .01***	125, .06, .49	125, .09, .33
**Homogeneous**	53, .13, .37	53, .01, .92	5, .10, .49	53, .16, .24
**Heterogeneous**	72, .33, < .01***	72, .34, < .01***	72, .03, .79	72, .03, .83

* *p* ≤ .10.

** *p* ≤ .05.

*** *p* < .01.

### Relationship between MEIM and college students’ implicit measures

In addition to completing the same implicit and explicit measures as the child participants, young adults completed the Multigroup Ethnic Identity Measure [MEIM; 10] to measure strength of ethnic identity. A significant positive relationship was observed between young adults’ scores on the MEIM and their implicit Black bias, *r* (125) = .15, *p* = .05. When looking at the subscale of belonging, we found a positive relationship between how strongly participants felt they belonged to their group and their implicit pro-Black over White bias, *r* (125) = .18, *p* = .02. Further, an independent sample t-test revealed that students who attended an all-Black college endorsed a stronger sense of belonging to their racial group, *t* (124) = 2.75, *p* < .01 and had higher total scores on the MEIM, *t* (124) = 3.11, *p* < .01, than did peers at the heterogeneous college.

## Conclusion

Researchers have long struggled to understand why African-Americans, on average, show no mean level implicit own group bias, when other groups frequently exhibit an implicit bias for their own group. Perhaps even more puzzling, studies reporting an absence of an own group bias among African-Americans frequently observe bias scores that are normally distributed around the midpoint, with approximately half the sample exhibiting a bias for their own group, and half exhibiting a bias for the outgroup [24–25; 40; 43; 45–46]. Our study provides one of the first comprehensive exploratory examinations of factors that may contribute to the presence (or absence) of implicit own group positivity (and identity) among African-Americans.

Replicating prior research [40; 43], our sample of African-American children showed, on average, no implicit bias for either their racial ingroup (Black) or their racial outgroup (White), despite exhibiting a strong explicit bias for their own group. Further, our finding that the racial composition of the schools tested (predominantly Black vs. racially mixed) did not predict implicit bias in our sample of African-American children is consistent with a sample of European-American youths using a very similar methodology [40]. Indeed, among children immersion in predominantly Black environments was not sufficient to lead to an increase in implicit own group bias.

Our sample of African-American young adults differed from the samples of previous studies, [24–25; 45–46] in that they exhibited a significant implicit ingroup preference. Notably, this finding was driven by students attending a Historically Black College. Similarly aged African-Americans attending a racially mixed college exhibited no mean level implicit bias for Black over White, consistent with past studies of African-Americans’ implicit race bias [24–25; 45–46]. Our finding of an implicit pro-Black bias at a Historically Black College aligns with previous research showing stronger *explicit* pro-Black bias in students from Historically Black Colleges than their counterparts at predominantly White colleges [51]. Indeed, our study is the first to demonstrate that the often generalized notion that African-Americans lack an implicit own group bias is not universally applicable to individuals who belong to culturally stigmatized groups. Instead, the previously reported lack of an implicit ingroup bias may represent a sampling bias that typically draws from African-Americans living in communities where they are part of the local minority.

There are two possible explanations for why we found that students attending a predominantly Black college expressed an implicit preference for Black over White: 1) more contact with Black people in positions of leadership and power at the college led to the formation of more pro-Black attitudes or 2) there was a self-selection bias and people with more pro-Black bias self-selected to attend the Historically Black College. More research is needed to adjudicate among these possibilities. Our data showing that regardless of college-type, those young adults who attended all-Black schools from K-12 had significantly higher implicit pro-Black over White bias than their counterparts who attended racially heterogeneous schools suggests that being in an all-Black environment *before* college, with people in positions of leadership and power, may increase African-Americans’ pro-Black implicit bias. While there were no differences in implicit bias based on school type for our child sample, observing a difference in our young adult sample based on the schools they attended prior to college suggests that prolonged schooling in an all-Black environment may be essential for the formation of a positive implicit own group bias. In order to develop a pro-Black implicit bias, African-Americans may have to remain in all-Black schools past childhood and into adolescence. This argument fits with previous research showing that exposure to positive Black exemplars increases positive implicit bias toward African-Americans among older (but not younger) children and adults [62–63].

In terms of explicit bias, African-American children, regardless of school type, overwhelmingly exhibited an explicit bias for their ingroup. Unlike previous research that reports stronger pro-Black explicit bias among African-American children from homogeneous schools than those from heterogeneous schools [49], our study shows comparable levels of pro-Black bias regardless of school type. Even among the young adult sample, we demonstrated an explicit preference for Black over White regardless of participants’ school composition. Replicating past research [51], we observed stronger explicit pro-Black attitudes and identity on the Multi-Ethnic Identity Measure among students from the Historically Black College than those from the racially heterogeneous college. The explicit pro-Black bias that we found in children and young adults, at least in part, reflects the fact that in the years since the Civil Rights and Black Power Movements, it has become more socially acceptable for African-Americans to display explicit positive ingroup bias (i.e., “I’m Black and I’m proud”) [41–42]. The increase in explicit ingroup positivity with age observed in African-Americans has been previously supported [21; 64], and stands in contrast to the pattern typical of European-Americans, who report a decrease in explicit ingroup positivity with age.

Turning to our identity measures, children and young adults strongly identified with their ingroup on implicit and explicit measures. Moreover, identification with the ingroup increased with age on both measures. This pattern is consistent with past studies showing that African-Americans’ explicit identification with Black over White becomes stronger with age [21; 64]. While this study is the first to examine the implicit race identity of African-Americans, our observation that implicit identification with the ingroup increases with age is consistent with past research in other minority populations [48].

Unlike previous research that has found no relationship between implicit bias and age [7–8; 24–25], our study revealed a non-significant trend toward a relationship, with young adults showing slightly stronger pro-Black over White implicit bias than children. Previous researchers have questioned how generalizable the relationship between age and implicit bias is for minority populations [8]. While it has been suggested that implicit biases are acquired via a fast learning model, in which they come online early in life and remain stable throughout the lifespan [7], the majority of this work has been conducted with participants from culturally high status groups. We are inclined to believe that the relationship between age and implicit bias may look different in a group of individuals who, from a very early age, are gradually accumulating racial socialization messages that either foster or combat the negative portrayal of their group in society.

Our study is in alignment with previous research showing that African-American’s implicit and explicit bias are related [45]. Interestingly, we only found a significant relationship between implicit and explicit measures in our young adult sample, particularly those attending the racially heterogeneous college. This suggests that balance in explicit and implicit bias/identity is achieved later in development (post-adolescence), and that this process is most likely to occur in social environments where the salience of racial group differences is high.

In terms of parental influence, our study supports previous research that has found parents’ transmission of racial socialization messages about cultural history and racial pride positively impact African-American children’s explicit racial identity [53–60]. Indeed, our study is the first to show that African-American parents’ attitudes are related to their children’s implicit race bias, much as European-American parents’ attitudes predict their children’s implicit bias [50]. While we found several relationships between parent and child measures, one of particular interest was the negative association—only observed among children attending all-Black schools—between the frequency of messages preparing children for bias and implicit race attitudes. Specifically, the more that parents of children attending racially homogeneous (all-Black) schools emphasized racism and attempted to prepare their children to face discrimination, the less their children exhibited a positive implicit bias for their ingroup. By contrast, a significant positive association between preparation for bias messages and explicit ingroup bias emerged for all children; the more parents emphasized messages about racism, the stronger children’s explicit preference for Black over White. Parents’ preparation for bias messages thus appear to influence biases in complex ways, likely in part because these messages convey a rich and varied set of information. Messages that one's ingroup is valuable despite being negatively stereotyped, for instance, are likely to bolster explicit ingroup bias. However, with preparation for bias messages, parents also often provide information that highlights the low social status of the ingroup and focus on the risk of harm from owngroup members. Information about the lower status of one’s ingroup and the threats they face from members of their group may foster negative implicit bias toward the ingroup. Consistent with this interpretation, past research with African-American adults has demonstrated that perceived negativity of the group relates positively to adults’ explicit ingroup bias, but negatively to their implicit ingroup bias [45].

One main difference between the present study and past research on African-Americans’ implicit bias is the geographical location in which this study took place. The majority of past work has focused on African-Americans in North-eastern residing in majority White mid-high socio-economic status regions of the United States. In contrast, our work was conducted in Atlanta, Georgia, a region in the south that has a large number of African-Americans of mid- to high socio-economic status. Atlanta has been labeled the “Black Mecca” due to its historically high concentration of Black wealth, political power, and culture. The city boasts an unusually large number of African-American millionaires [65] and has been singled out as a location that is conducive to economic success among African-Americans [66]. Atlanta also has numerous Historically Black Colleges and Universities that have attracted and educated a large population of local African-Americans who have achieved high academic and social status [67]. Participants in the present study, particularly those who attended predominantly Black schools, were thus immersed in a social context that reinforces ingroup positivity. Our data suggest that we need to reconsider our tendency to think about intergroup bias as monolithic and instead need to consider the environmental factors associated with individual differences in observed race bias.

## Supporting information

S1 File Young adult sample. Raw data for all young adult sample analyses.
(SAV)

S2 File Child sample. Raw data for all child sample analyses.
(SAV)

S3 File All participants combined. Raw data for all age group comparison analyses.
(SAV)
